# Transcriptomic Landscape of Herbivore Oviposition in Arabidopsis: A Systematic Review

**DOI:** 10.3389/fpls.2021.772492

**Published:** 2022-01-21

**Authors:** Dairon Ojeda-Martinez, Isabel Diaz, M. Estrella Santamaria

**Affiliations:** ^1^Centro de Biotecnología y Genómica de Plantas, Universidad Politécnica de Madrid – Instituto Nacional de Investigación y Tecnología Agraria y Alimentación, Madrid, Spain; ^2^Departamento de Biotecnología-Biología Vegetal, Escuela Técnica Superior de Ingeniería Agronómica, Alimentaria y de Biosistemas, Universidad Politécnica de Madrid, Madrid, Spain

**Keywords:** Arabidopsis, eggs, herbivore, oviposition, Pieris, systematic review, Tetranychus

## Abstract

Herbivore oviposition produces all sorts of responses in plants, involving wide and complex genetic rearrangements. Many transcriptomic studies have been performed to understand this interaction, producing a bulk of transcriptomic data. However, the use of many transcriptomic techniques across the years, the lack of comparable transcriptomic context at the time of publication, and the use of outdated databases are limitations to understand this biological process. The current analysis intends to retrieve oviposition studies and process them with up-to-date techniques and updated databases. To reduce heterogeneities, the same processing techniques were applied, and Arabidopsis was selected to avoid divergencies on plant taxa stress response strategies. By doing so, we intended to understand the major mechanisms and regulatory processes linked to oviposition response. Differentially expressed gene (DEG) identification and co-expression network-based analyses were the main tools to achieve this goal. Two microarray studies and three RNA-seq analyses passed the screening criteria. The collected data pertained to the lepidopteran *Pieris brassicae* and the mite *Tetranychus urticae*, and covered a timeline from 3 to 144 h. Among the 18, 221 DEGs found, 15, 406 were exclusive of *P. brassicae* (72 h) and 801 were exclusive for the rest of the experiments. Excluding *P. brassicae* (72 h), shared genes on the rest of the experiments were twice the unique genes, indicating common response mechanisms were predominant. Enrichment analyses indicated that shared processes were circumscribed to earlier time points, and after 24 h, the divergences escalated. The response was characterized by patterns of time-dependent waves of unique processes. *P. brassicae* oviposition induced a rich response that shared functions across time points, while *T. urticae* eggs triggered less but more diverse time-dependent functions. The main processes altered were associated with hormonal cascades [e.g., salicilic acid (SA) and jasmonic acid (JA)], defense [reactive oxygen species (ROS) and glucosinolates], cell wall rearrangements, abiotic stress responses, and energy metabolism. Key gene drivers of the identified processes were also identified and presented. The current results enrich and clarify the information regarding the molecular behavior of the plant in response to oviposition by herbivores. This information is valuable for multiple stress response engineering tools, among other applications.

## Introduction

The interaction between plants and arthropod herbivores is a complex molecular event where both parts try to ensure their survivability. Plants use cues emitted by herbivores such as sex pheromones (Helms et al., 2017) or even volatiles from neighboring damaged plants (Karban et al., 2014; Pashalidou et al., 2020), and prepare their defenses for future attacks. Among these cues, herbivore eggs induce several defense responses in many plant species (Gouhier-Darimont et al., 2013; Fatouros et al., 2014; Bittner et al., 2017; Geuss et al., 2017). These include callus formation, necrosis, volatile blend alterations, or accumulation of ovicidal compounds (Stahl et al., 2020). All of these morphological manifestations involve a wide genomic rearrangement in response to this seemingly harmless life stage. Several studies have delved into the transcriptomic analysis of this response, indicating major pathways and events (Firtzlaff et al., 2016; Nallu et al., 2018; Lortzing et al., 2019; Stahl et al., 2020). Egg-induced alterations of the plant genetic makeup have been studied in highly diverse plant taxa, ranging from angiosperms to gymnosperms or from short-lived herbaceous species to long-lived trees. For a deeper analysis of them, the reader is encouraged to consult reviews such as Hilker and Fatouros (2015) or the research by Lortzing et al. (2020). Since plant stress response involves molecular events of high complexity (Vandereyken et al., 2018), the response to oviposition is also expected to be elaborated, as the aforementioned studies suggest. Therefore, to get a better understanding of these sophisticated chemical and molecular mechanisms, the usage of omic approaches is advised. Transcriptomic techniques provide a suitable option. Among the existing omic alternatives, transcriptomics has had a fast evolution compared to its counterparts, allowing the easier monitorization of massive amounts of data (Mosa et al., 2017). They ease the understanding of molecular events involved in biological processes (BPs) using the information of gene function and structure. It is a vehicle that helps to move beyond broad pathways and processes to uncover the molecular determinants of a certain stress. Along with such a powerful tool as transcriptomics, a methodology capable of generalizing the breadth of information generated becomes important as well. Gene co-expression networks can be used for such purpose. They can detect hubs of genes associated to BPs, highlight gene drivers of a transcriptional response, and indicate which genes are active simultaneously (Van Dam et al., 2018).

Due to the increasing application of transcriptomics to oviposition, the amount of data is growing although it is still limited. The analysis of the available transcriptomic data expands the precise conclusions and significance of the individual studies. Enhancing the interoperability of the available transcriptomic datasets contributes to the elucidation of the structure of complex biological systems. Several hurdles arise when performing this task, being the diversity of herbivores and plants involved one of them, which complicates the unveiling of general processes. Not all plants have the same amount of genetic information available in databases. Moreover, the quality of the available genome assemblies and their associated annotation data change largely between plant species. The gaps in this knowledge may induce the loss of important associated events and could indicate non-existent differences (Chaw et al., 2019; Zhang et al., 2021). Also, the fast evolution of transcriptomic processing techniques introduces heterogeneities when comparing results over the years. The improvement of microarray and RNA-seq technologies greatly enhances the mapping process and thus the quality of derived results. Therefore, the use of diverse transcriptomic methods could induce both the loss of information or introduce false-positive data (Yi et al., 2018). Gene information and association to ontologies is likewise an evolving area, which sometimes deprecates some of its reported data (The Gene Ontology Consortium, 2021). Additionally, most of the studies at the time of publication lacked a transcriptomic context to allow accurate analysis and comparisons.

The current study generalizes and presents the pathways altered during arthropod pest egg contact with plants and their key gene drivers. To do so, we focused on Arabidopsis, given the availability of a vast amount of genetic information for this species. By selecting oviposition studies only on this plant, we controlled the heterogeneity of transcriptomic data available. We reanalyzed raw transcriptomic data using state-of-the-art software and the latest versions of annotation databases, adding fresh light on the valuable transcriptomic information. Processing all the available data with the same techniques enabled us to control heterogeneities also on the original analyses caused by data processing. Moreover, the presence of transcriptomic information on multiple data points and from different species allowed comparisons and inquiries that expanded the original interpretation. Data were further explored and summarized by means of network tests that also allowed the identification of co-expressed gene hubs and key gene drivers.

## Methods

### Data Collection and Curation

The data selection process occurred on three main platforms: the sequence read archive (SRA) repository, gene expression omnibus (GEO) database, and paper-wise through Google Scholar. SRA and GEO were queried by using the keywords “eggs” or “oviposition” for RNA assays and restricted to *Arabidopsis thaliana* studies. A total of 500 studies were screened on the aforementioned platforms. Google Scholar search for papers related to oviposition analyses was restricted to the years 2000–2021. Keywords used were “Pieris AND Arabidopsis AND egg,” “RNA AND herbivore AND oviposition,” and “RNA AND herbivore AND oviposition OR egg extract OR egg.” A further search focused on authors known to perform oviposition studies was also pursued. For each year, 15 pages containing 10 articles each were searched, for a total of 3,150 articles manually screened.

The selection criteria for the studies to be included comprised: oviposition and/or egg extract experiments, the performance of transcriptomic analysis (RNA-seq/Microarray), the plant subject being *A. thaliana* (Col-0), experiments with clear and separated oviposition stress versus control replicates, of which only samples taken without any other stress were selected. Also, the availability of the raw data to be processed was of importance during the selection. Data were searched and downloaded from May to August 2021.

RNA-seq datasets were analyzed by using the R packages Sleuth (ver. 0.30.0) (Pimentel et al., 2017) and Kallisto (ver. 0.46.1) (Bray et al., 2016). Adapters and poly-N sequences were removed from raw data as well as low-quality reads and rRNAs. Quality checks were done using the FastQC toolkit (Andrews, 2010) and trimming and filtering were performed using the default settings of Trimmomatic (Bolger et al., 2014) and SortMeRna (Kopylova et al., 2012). Raw data management by Kallisto was done according to default settings, bootstrapping samples 100 times. Normalization of both estimated fragment count and transcript per million (TPM) values was performed using Sleuth. To reduce batch effect across experiments, the normalization processes performed by Sleuth were done on each experiment individually, which allowed data management according to experiment-specific characteristics. The reads were mapped to the Arabidopsis TAIR10 genome release using Sleuth (ver. 0.30.0). Bootstrap information helped in the reduction of the technical variance during the abundance estimation of each gene. Samples were screened for outlier presence by analyzing principal component analysis (PCA) plots and heatmaps, both at individual and across experimental levels. When a potential outlier was spotted, non-parametric modeling of the data excluding the sample was performed, and residual sum of squares (RSS) reduction was evaluated in order to determine its elimination from the analysis.

### Differentially Expressed Gene Identification and Functional Analysis

Differentially expressed genes (DEGs) were calculated based on fold change (FC) and *p*-values obtained using Sleuth. Each treated sample was compared to its control through a Wald test, and the Lancaster aggregation method rendered the final DEG list (Yi et al., 2018). Differential expression was considered when a gene’s Lancaster aggregated *p*-value was *p* < 0.05. DEG list depictions using Venn diagrams were constructed on the free online resource InteractiVenn (Heberle et al., 2015). Regarding the microarray information processing, the raw data of both experiments were independently normalized using the R package limma (Ritchie et al., 2015). A Benjamini and Hochberg *p*-value adjustment was performed to identify DEGs. All of the aforementioned processes were performed on the GEO2R online platform (Barrett et al., 2012) of the NCBI online website (Sayers et al., 2021). Only the samples identified as controls and egg extract/oviposition-treated were processed.

A Gene Ontology (GO) enrichment analysis was performed on the DEGs identified using the g:Profiler web server (Raudvere et al., 2019), excluding the electronic annotations. The GO categories corresponding to BPs were introduced in Cytoscape for further analysis according to Reimand et al. (2019). The EnrichmentMap app (Merico et al., 2010) from Cytoscape was used to visualize as a network/map the GO results obtained for each study. The aforementioned GO networks were then processed by the AutoAnnotate app from Cytoscape to identify clusters of similar terms representing major BPs.

### Network Construction and Processing

A global protein–protein interaction (PPI) network was assembled by merging experimentally verified PPIs collected from the TAIR (Lamesch et al., 2012), IntAct (Kerrien et al., 2012), and BioGRID (Oughtred et al., 2019) databases using Cytoscape (ver. 3.8.2) (Shannon et al., 2003). The aforementioned process allowed to gather as much experimental information as possible regarding PPI events, containing the resulting network 17,422 nodes and 156,464 edges. This network was used to identify the direct interactors of the list of DEGs. For that purpose, first neighbor nodes and adjacent edges of the list of DEGs were extracted to construct individual networks for each experiment. Links to the assembled PPI networks and a file containing the steps followed to construct them can be found at the end of this work (see section “Data Availability Statement”).

### WGCNA-Based Analyses for Differentially Expressed Genes and Interactors

Differentially expressed genes and their interactor’s analyses continued by using the R package weighted correlation network analysis (WGCNA) (version 1.70) (Langfelder and Horvath, 2008). Pair-wise gene expression Pearson correlation (PC) and Euclidean distance were calculated for each sample to generate a similarity matrix. Afterward, an adjacency matrix was constructed for each list, using the approximate scale-free topology criterion to select the soft thresholding power. The cutoff criteria were ≥15 genes and cut height = 0.25, corresponding to modules having eigengenes with a correlation of 0.75 or higher. A Topological Overlap Matrix was then constructed, to reduce the effects of noise and spurious associations. List-specific co-expression networks were then calculated, and co-expression modules were identified. For the identification of modules, soft threshold powers were selected for each study according to the preconditions of approximate scale-free topology (Supplementary Table 1). The eigengenes of each identified module were used to detect a significant association to the stress response of each analysis. The modules significantly correlated to the stress (Pearson’s *r* ≥ 0.9 and *p* < 0.05) and having significantly associated genes to oviposition traits were selected for further analyses. Gene co-expression networks were constructed based on the WGCNA results and exported and visualized on Cytoscape (Shannon et al., 2003). The plugin Network Analyzer of the Cytoscape software was used to calculate network topological characteristics and statistics. The identification of the node degree distribution was confirmed through a regression analysis performed using the R software (R Core Team, 2021). Regression significance was determined when *p*-values were *p* < 0.05.

### Gene Ontology Enrichment Analysis

The modules correlated with the oviposition traits, containing DEGs and co-expressed interactors, were subjected to a GO enrichment analysis and kyoto encyclopedia of genes and genomes (KEGG) pathway analysis. Analyses were performed on the Metascape online tool^1^ (Zhou et al., 2019). Significance for each analysis was determined under the criteria of *p* < 0.05. Analysis of the superposition of the gene lists per module and shared functions were performed as well *via* Circos plots in Metascape. A heatmap that compared and clusterized the modules based on their BP behavior was also constructed *via* Metascape.

## Results

### Data Collection and Processing

**FIGURE 1:**
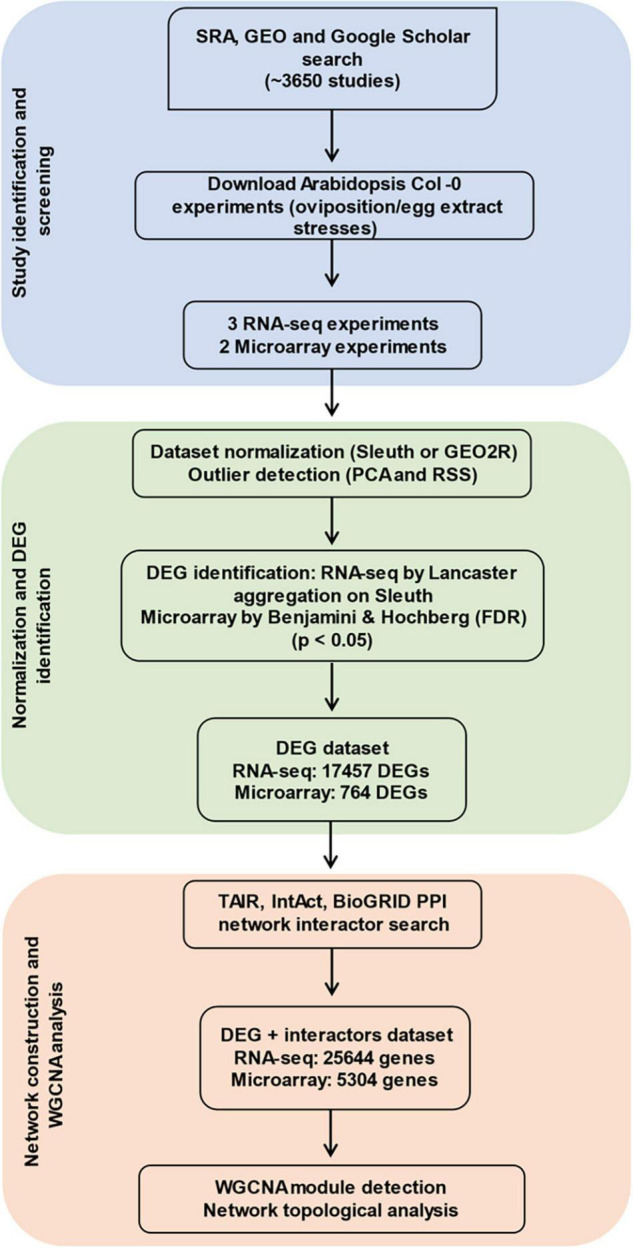
Workflow for data collection, curation, and co-expression network analysis. In total, five different studies passed the filter and were analyzed by identifying differentially expressed genes (DEGs) from them. Experiment-based interactors of the DEGs were searched from databases, and co-expression based on each experimental data was determined. Significant groups of genes plus interactors (modules) were obtained and analyzed.

A summary of the analysis process is described in Figure 1. The main strategies following data collection were the prediction of DEGs, construction of networks, significant module detection, and ontology characterization. Further details on the identification of studies *via* databases and other methods were collected in a PRISMA flow diagram (Supplementary File 2).

**FIGURE 2:**
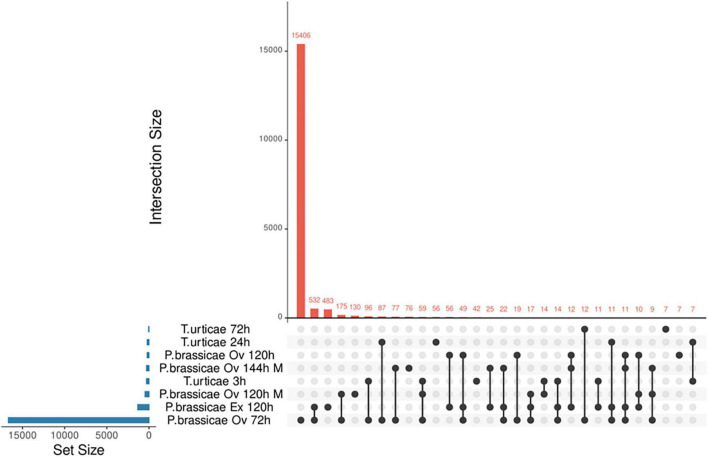
Upset diagram of unique and common DEGs found in *A. thaliana* under oviposition or egg extract stresses. Each study is identified by the pest name, followed by the identification of the stress: oviposition (Ov) or egg extract (Ex) and the time of analysis (h). All *T. urticae* data were obtained from egg extract stress. Microarray results are identified by an (M), the rest of the results were obtained from RNA-seq data.

From the screening process, five studies passed the selection criteria. Two microarrays (GSE114041 and GSE69623) and three RNA-seq analyses (SRP244078, SRP134094, and GSE168993) were retained (Supplementary Table 2). The microarray studies were done on local plant tissue whereas the RNA-seq studies covered local and systemic responses. The studies SRP134094, SRP244078, GSE114041, and GSE69623 used the lepidopteran *P. brassicae* eggs/egg extract as stressors and the study GSE168993 used the spider mite egg extract. Pieris-related analyses covered from 72 to 144 h, while the Tetranychus-related study ranged from 3 to 72 h. All experiments from *Tetranychus urticae* were performed using egg extract, whereas those of *P. brassicae* involved egg extract or oviposition. Studies were identified by the pest’s name, the time at which the samples were taken and whether egg extract (Ex) or oviposition (Ov) was used. A total of 17,457 DEGs were identified for the three RNA-seq studies and 764 DEGs for the microarray data. Here, we report the number of genes unique and shared by each phytophagous species at each time point evaluated (Figure 2 and Supplementary Figure 1). The species and time presenting the larger number of unique DEGs was *P. brassicae* at 72 h (Figure 2). The lowest unique DEG number was shared between the latest time point of the *T. urticae* data and *P. brassicae* at 120 h. A file containing the detailed list of the unique DEGs by study can be consulted on Supplementary File 3. The dynamics of the genes upregulated and downregulated can be consulted on Supplementary Figure 2A. FC expression 10 times over controls was rare (Supplementary Figure 2B), or not present as in the microarray data. DEGs expressed up to three times over their controls were generally the most common.

### Functional Analysis of Differentially Expressed Genes

**FIGURE 3:**
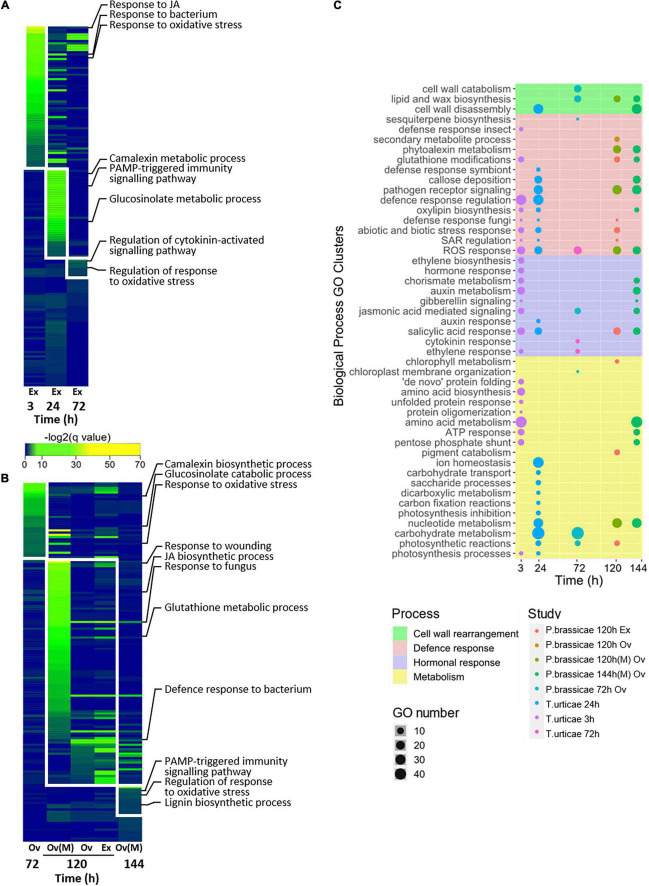
Identification and clusterization of the main BPs across experiments. **(A)** Waves of time-specific BPs identified for *T. urticae*. **(B)** Waves of time-specific BPs identified for *P. brassicae*. **(C)** Clusterization of the similar biological processes (BPs) at each time point. The names of the clusters are shown and the major processes to which they belong are represented as different colors. Species and time (h) are stated for each study; stresses are identified as Ov (oviposition) and Ex (egg extract). All *T. urticae* data were obtained from egg extract stress. To represent **(A,B)**, a log transformation of the corrected *p*-values of the BP enrichments was used. Microarray results are identified by an (M), the rest of the results were obtained from RNA-seq data. Enrichment was calculated using an over representation analysis (ORA) test with Benjamini–Hochberg correction [false discovery rate (FDR) ≤ 0.05].

Enrichment analysis was performed on the lists of DEGs and similar BPs were clusterized (Figures 3A–C). Time-dependent patterns of enriched BPs were revealed as progressive waves of unique events (Figures 3A,B). Clusterized mechanisms could be further grouped into metabolism, defense, cell wall rearrangement, or hormonal processes (Figure 3C). Early responses to *T. urticae* egg extract were associated with amino acid and JA metabolism, as well as negative regulation of oviposition. Later events were enriched on amino acid, ion, and carbohydrate metabolism as well as negative regulation of responses. Some of the most important defense responses found for *T. urticae* were associated with JA, glutathione, glucosinolates, and callose deposition. On the other hand, *P. brassicae* had at 72 h processes enriched on cell wall reorganization and later on, secondary metabolite alterations and regulation of pigment metabolism. The processes common to all studies were associated with ROS, glucosinolate metabolism, pathogen defense responses, and cell wall modifications. Unique clusters of BPs were observed at 24 h of the response to *T. urticae*. Unlike this behavior, the functional response to *P. brassicae* was scarcer on unique clusters, sharing most of the GOs with other time points and studies. A detailed picture of the enriched BP behavior across the experiments and their clusterization can be consulted in Supplementary Figures 3, 4, respectively.

### Construction of Co-expression Networks Using Oviposition Differentially Expressed Genes

The DEG lists originated from each study were used to search for their interactors in a PPI network. By applying this method, not only direct interactions among the proteins coded by the DEGs could be unraveled, but also indirect interactions through intermediaries. This information clarifies the molecular environment in which the DEG products participate, expanding the biological significance of simple gene lists. Using the constructed network that contained experimentally verified PPIs, the potential DEG interactors were identified. Correlations between the DEGs and their interactors were then calculated for each study independently using gene expression. Clusters of highly correlated proteins (modules) were identified, which were then used to construct a PPI network for each study.

The biological significance of the constructed networks and modules was assessed. The biological networks are usually arranged in modules, their node degrees follow a power law distribution and the modules present enriched functions (Jiang et al., 2016; Wang et al., 2020). To verify these properties, a series of tests including topological, modularity, and functional enrichment analyses were performed. All the constructed networks presented topological properties similar to typical biological networks. The behavior of the node degrees following a power-law distribution was confirmed (Supplementary Figure 5). Network topological parameters reflecting the general arrangement of nodes or internal interactions are displayed in Supplementary Table 3. The biological networks are also commonly organized into modules of closely connected nodes of co-expressed genes. To verify modularity, hierarchical clustering and branch cutting were performed on each study data. A total of 178 modules were identified across the experiments (Supplementary Table 1), confirming the PPI network modular behavior. To further verify the biological significance of the modules, correlation to the oviposition traits was verified. A total of 18 modules were correlated to oviposition processes (*r* > 0.9, *p* < 0.05), which encompassed 8,742 genes. A full list of the significant modules by study, the number of genes they contained, and correlation coefficients concerning oviposition/egg extract can be consulted in Supplementary Table 4.

**FIGURE 4:**
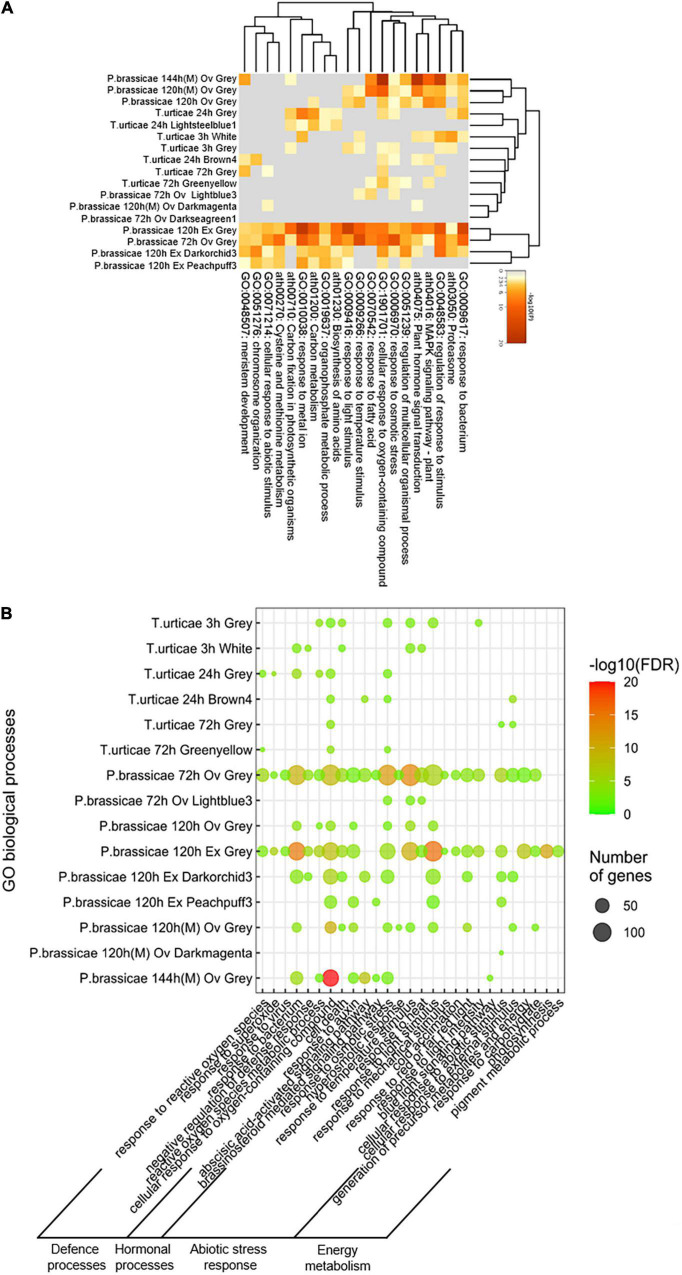
Gene enrichment analysis of the BP ontologies for each module of co-expressed genes. **(A)** Heatmap of the enriched ontologies across modules, colored by *p*-values. **(B)** Representation of the four groups that formed the top enriched terms across the experiments. Species and time (h) are stated for each study; stresses are identified as Ov (oviposition) and Ex (egg extract). All *T. urticae* data were obtained from egg extract stress. Microarray results are classified by an (M) at the end of the identification, the rest of the results were obtained from RNA-seq data. Enrichment was calculated based on Benjamini–Hochberg tests (FDR ≤ 0.05).

The biological significance was also verified by applying enrichment analysis on the modules identified. Genes belonging to the modules correlated to the stress and their ontologies are shown in a Circos plot (Supplementary Figures 6A,B). Gene overlapping was more common on the response to *T. urticae* than to *P. brassicae*. However, the majority of the exclusive genes of the latter performed functions common to other time points, unlike the exclusive genes of the former (Supplementary Figure 6B). A heatmap analyzing the associations of the studies in terms of enriched ontologies is displayed in Figure 4A. Most of the Pieris results were clustered together in two groups at the margins of the plot. Similarly, all of Tetranychus modules were tightly associated. Two modules from Pieris at 72 h (Lightblue3 and Darkseagreen1) and one from the microarray at 120 h (Darkmagenta) were clustered closer to the plant response to Tetranychus at 72 h. Among the enriched BPs for the aforementioned modules at 72 h were cellular response to abiotic stimulus, response to fatty acid and response to osmotic stress. Four main groups of functions were found across all the BPs (Figure 4B). Among the defense terms, bacterial and ROS responses were the most frequent. Hormonal and abiotic stress responses were also detected, as well as multiple processes related to energy metabolism. At 72 h (the common time point among the two species), the modules of the response to Pieris had functions enriched across the four main groups. On the case of the response to Tetranychus, the enriched processes were scarcer and there was no enriched function on the hormonal process group. After assessing the biological significance of the constructed PPI networks, we focused on the identification of the genes regulating the oviposition stresses.

### Identification of Key Regulators

**FIGURE 5:**
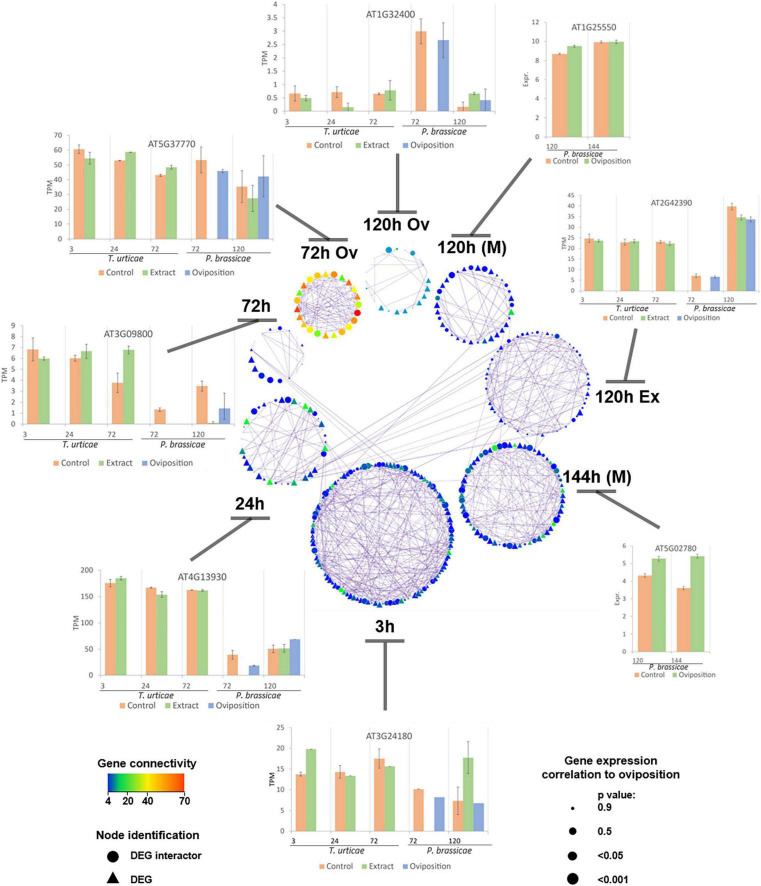
Protein–protein interaction network representing DEGs and interactors from the modules correlated to the oviposition stress. Co-expression among components of the current network is identified by lines (edges). A representation of the transcript per million (TPM) behavior of some of the key regulators per experiment is depicted. Columns represent means and bars the standard error of the mean (SEM). Node size increases when the *p*-value of the correlation between the gene and the experiment decreases. Color indicates connectivity, being red more connected and blue less connected genes. Genes having less than three connections were not represented on this network. Nodes represented as triangles correspond to DEGs and circles to their co-expressed interactors. Each time point represents the results of a study, from the bottom and clockwise: 3–72 h, *T. urticae* egg extract; 72 h Ov, *P. brassicae* oviposition; 120 h Ov, *P. brassicae* oviposition; 120 h (M), *P. brassicae* oviposition; 120 h Ex, *P. brassicae* egg extract; 144 h (M), *P. brassicae* oviposition. Microarray results are identified by an (M), the rest of the results were obtained from RNA-seq data.

Key regulators of the modules were identified based on network parameters and statistical analyses. Several characteristics were used such as the number of genes with which they co-expressed, the strength of the co-expression, inter-hub centrality, the gene correlation to the stress of interest, and their TPM expression value. The significant modules were merged in a single network (Figure 5) for visualization purposes, keeping genes that had more than three co-expressors. *P. brassicae* had only two time points that presented unique pairs of co-expressed genes (oviposition at 72 and 120 h). The results of *T. urticae* at 3 h were the only ones that shared co-expressed interactions with all the modules. In terms of connection, Pieris at 72 h had the most connected genes among all the studies and also the least connected components at 120 h (regardless of stress). Tetranychus had genes correlated to oviposition at all its time points, whereas Pieris had them only at 72 and 144 h. Generally, the correlated genes had also a higher number of connections among the modules. A representation of the TPM behavior of some of the key genes per study and time point was depicted in Figure 5.

A wide array of functions was covered by the key regulators of the two herbivore species. At 3 h, some of the *T. urticae* regulators were related to plant–pathogen interaction (the phosphatase-like SGT1A, AT4G23570, and the BRI1-associated receptor kinase BAK1, AT4G33430), biosynthesis of secondary metabolites (the lipoamide dehydrogenase LPD2, AT3G17240 and the dihydrolipoamide succinyltransferase AT4G26910), and lipids (the glucosylceramidase AT3G24180). The two last time points of this species had genes related to glutathione and secondary metabolism (the microsomal glutathione s-transferase AT1G65820; the phosphoglycerate kinase PGK1, AT3G12780; the serine hydroxymethyltransferase SHM4, AT4G13930; the tryptophan synthase TSB2, AT4G27070), transcription-related such as ATRSP41, AT5G52040, and the dewaxing AP2/ERF-type TF (ERF107, AT5G61590), and a SNARE vesicular protein (AT3G09800). The highly interconnected genes of Pieris at 72 h were associated to endoplasmic reticulum (ER)-based SAR responses (the ER oxidoreductin AERO1, AT1G72280, and the SecY protein AT2G34250), plant–pathogen interaction (the calmodulin-like CML24, AT5G37770), or mitochondrial processes (the NFU domain-containing protein NFU4, At3g20970). At later time points, *P. brassicae* oviposition correlated genes were associated to glutathione metabolism (the glutathione transferase GSTL1, AT5G02780), hormonal signal transduction (the GRAS transcription factor GAI, AT1G14920), apoptosis regulation (the apoptosis suppressor DAD1, AT1G32210), biotic signal reception, and response by LRRs or kinases (the LRR AT3G02880; the kinase AT2G42390; and the transmembrane protein TOM2A, AT1G32400) and TFs such as HHO3, AT1G25550. A list of the main regulators of the networks constructed, their network statistics, and PC data can be consulted in Supplementary Table 5.

## Discussion

### Differentially Expressed Genes and Their Regulation Across Experiments

As a result of our reanalysis, a comprehensive list of the unique DEGs per time point and species was produced. Of the 18,221 DEGs found for RNA-seq and microarray experiments, 15,406 were exclusive of *P. brassicae* (72 h) and 801 were exclusive for the rest of the experiments. On the other hand, experiments shared 2,014 DEGs, which were more than twice the unique genes (excluding 72 h *P. brassicae* DEGs). This result suggested a transcriptional resemblance of the Arabidopsis response to the oviposition by both pests. On this note, a limitation of the present study was that there was information of the plant response for both pests for a single time point. Due to this characteristic, we redirected our focus toward a functional comparison regardless of time. The analyses were done accounting for the time frames covered by the original assays for each pest species. Similar transcriptomic responses to oviposition across different plant species have been previously reported (Bertea et al., 2020). Some described similarities were highlighted in our results such as ROS, defense, and metabolic processes. The presence of exclusive genes was also expected when divergences such as oviposition strategies or pest taxa, etc., are considered. Concerning transcriptomic species specificities, no gene was commonly altered on *T. urticae* data, while five genes were altered at all *P. brassicae* time points. These genes included two receptors, a gibberellin receptor (*GID1B*, AT3G63010) and a receptor-like protein (*RLP7*, AT1G47890); and three enzymes (*GPAT5*, AT3G11430; *PXMT1*, AT1G66700; and *SAG13*, AT2G29350). Interestingly, the gene *RLP7* belongs to a Ve-like family of genes. Members of this family are activated and participate in the resistance against highly aggressive isolates of the fungi *Verticillium dahlia* (Zhang et al., 2012). One of the main contributions of the current work was the identification of species- and time-specific genes during the plant response to oviposition, which can be consulted on Supplementary File 3.

The resemblance on transcriptomic response extended as well toward FC behavior and regulation. The regulatory processes were characterized by the domination of activated genes versus repressed. Transcriptomic responses in which activated genes dominate over the repressed are common within biotic stresses. Stimuli originated from nematodes (Atkinson et al., 2013), pathogens (van de Mortel et al., 2012), oviposition (Lortzing et al., 2020), or herbivory (Ehlting et al., 2008; Santamaria et al., 2021) are some of the examples where plants respond by displaying this imbalance. Gene activation has a high energetic cost due to the involvement of multiple mechanisms (Wray et al., 2003). On the other hand, repression processes are simpler and can occur by many ways (Chen and Rajewsky, 2007). Therefore, the domination of gene activation during oviposition stress regardless of its high cost suggests its evolutionary importance to plants. Regarding the intensity of the gene regulation, the plant response to *T. urticae* was dominated by values of FC < 3. Unlike this, *P. brassicae* response was mixed between the three reported FC ranges. The microarray results were also somehow divergent from the RNA-seq data. The differences were most likely associated with the divergence in the transcriptional scope of both analyses. The limited set of genes detected by microarrays introduces skews in the identification of differentially enriched gene families. Also, they could be due to samples being taken from local tissue on the microarray and not whole leaves as for the RNA-seq studies.

### Timeline of Functional Processes

A time-dependent pattern of unique waves of enriched BPs was identified for each species. Most of the involved BPs were related to defense mechanisms regardless of time, species, or stress. As early as 3 h, many plant strategies to counter oviposition were already in motion. These included ROS-responding genes (Reymond, 2013; Geuss et al., 2017), pathogen-triggered mechanisms, and JA-mediated hormonal processes (Hilker and Meiners, 2011; Hilker and Fatouros, 2015). Of them, the involvement of JA was exclusive of the response to the spider mite. In the middle time points of both species (24–120 h), defense functions involving glucosinolates, camalexin, and PAMP-triggered immunity were active. Our results indicating the relevance of pathogen defense responses agree with previous reports (Gouhier-Darimont et al., 2013; Reymond, 2013; Bertea et al., 2020). These molecular events deployed to counter *P. brassicae* oviposition/egg extract manage to inhibit the growth of multiple *Pseudomonas syringae* strains (Hilfiker et al., 2014). Whether the negative impact on bacteria would be the same when Arabidopsis is exposed to *T. urticae* egg extract, could be an interesting question to be answered. Later hours of the response to the lepidopteran stress revealed regulatory processes, most likely directing the metabolism of the plant toward homeostasis. Moreover, many of the described events such as ROS and glucosinolate production have been associated to herbivory responses (Nallu et al., 2018). The interaction of oviposition and herbivory has been explored. In the case of the lepidopteran, the pre-exposition to eggs or egg extract has had mixed results on the feeding pest stage (Gouhier-Darimont et al., 2013; Lortzing et al., 2019). Although the plant pre-exposition to egg extract in the case of the spider mite has stimulated the feeding behavior of the adults (Ojeda-Martinez et al., 2021). The aforementioned evidence could indicate a priming effect of the oviposition stress, which either warns the plant or tricks its defenses to enhance the success of the feeding pest.

Wide metabolic rearrangements were observed at all the time points of the studies. On Tetranychus-associated assays occurred a switch from amino acid and protein metabolism at 3 h, to photosynthetic and carbohydrate metabolism after 24 h. Pieris also showed at 72 h photosynthesis and carbohydrate alterations, followed by nucleotide metabolism adjustments. Photosynthesis and carbohydrate alterations are known plant responses to oviposition (Schröder et al., 2005; Velikova et al., 2010). However, their role in this response remains unexplained. The function of nucleotide metabolism changes due to arthropod oviposition remains as well unclear. Although, it could involve the activation of nucleic acid repair mechanisms or the use of nucleotides as signaling molecules or as metabolic intermediaries (Büchel et al., 2012; Das et al., 2021). An interesting response was found related to cell wall alterations on both species, especially toward later time points. Rearrangements such as disassembly, wax production, or lignin biosynthesis were some examples. Cell wall modifications occur during defense responses (Rui and Dinneny, 2020), and they are regarded as both an immediate defense line, or a more permanent measure that guards plants of future attacks (Miedes et al., 2014; Paniagua et al., 2017). Pieris-induced cell wall rearrangements were characterized by permanent mechanisms such as lignin and wax production. On the other hand, *T. urticae* directed its metabolism only toward cell wall disassembly processes. The generalization and comparison of the metabolic routes occurring during the plant response to pest oviposition is a contribution of the present work. Also, most of the previous studies on oviposition have focused on defense mechanisms. Our work expanded and updated the transcriptomic information beyond the classical defense scope.

### Gene Co-expression Network Analysis

Co-expression network analysis allows the integration of transcriptome data and PPI networks. It clarifies the molecular environment that influences or is being influenced by genes deemed as DE (Van Dam et al., 2018). Using this analysis, groups of densely interconnected genes are unveiled. These groups are active in the same BPs and allow to highlight regulatory genes during a stress response. During our study, PPI subnetworks were constructed using the DEG transcriptional data of each assay, integrating them into a three database PPI network. Hubs of genes significantly correlated to oviposition at each time point were identified. This allowed the understanding of first, the PPI environment of the DEGs, and second, with which proteins the DEG products were co-expressed. Transcriptomic dynamics occurring on Arabidopsis during oviposition were also clarified by analyzing the identified modules. The unveiling of the PPI environment of the DEGs active during the response to oviposition is another major contribution of the present study. Also, for the first time, an analysis on candidates to key regulators of the oviposition response is explored and presented; thanks to the performed generalization approach.

A peculiar behavior of the modules of *T. urticae* was that of sharing a very little number of enriched BPs with the rest of the analyses. Opposite to this, the results for Pieris announced a high number of common functions. The presence of unique genes and non-repeated functions happened mainly after 24 h for the spider mite. Six of the eight shared functions were limited to its earliest time point (3 h). The heatmap constructed from the module data confirmed the aforementioned functional discrepancy among both species. Most of the Pieris modules were functionally associated among themselves and separated from the Tetranychus data. The previous information underlined also the temporal variation of the transcriptomic response to oviposition. Indicating that Arabidopsis initial reaction to the oviposition of both pests is similar, but after 24 h it diverges. In addition, Pieris induces a response characterized by the use and recycling of functions, while the spider mite egg extract induces mostly heterogeneous and unique transcriptomic processes. The BPs of the modules were grouped in four categories: associated with hormones, energy metabolism, defense, and abiotic stress. The response to *P. brassicae* across these processes was broad and scattered. The events were particularly rich in hormonal processes and involved many energy-coupled functions. On the contrary, *T. urticae* egg extract induced a response focused on defense and abiotic stress mechanisms.

Additional information derived from gene hubs that helped to clarify the underlying behavior of the plant response was gene connectivity. *P. brassicae* eggs induced the expression of highly connected genes (up to 67 connections) unlike *T. urticae* (up to 15 connections). The high level of gene connectivity under Pieris egg stress could explain its abovementioned broader response. Along with connectivity, the rest of the modular analysis would induce to look into the original stimuli to understand Arabidopsis transcriptional variations. The divergence of the plant response to the oviposition of both pest species could be found in the identity of the elicitors to which it is reacting. According to Hilker and Fatouros (2015), differences in species-specific egg elicitors or on oviposition processes tend to induce different responses by plants. Recently, phosphatidylcholines (PCs) contained on *P. brassicae* eggs were identified as responsible for most Arabidopsis reactions (Stahl et al., 2020). The application of PCs on plants triggered SA accumulation and immune responses similar to natural oviposition. Unlike its counterpart, *T. urticae* egg extract involved JA cascades, among other processes (Ojeda-Martinez et al., 2021). This might lead to think that species-specific elicitors could be interacting with the plant, of which *P. brassicae*’s are undoubtedly inducing a more robust response. Nonetheless, the stresses by both species share their negative impact on the processes involving future generations. Pieris oviposition activates defenses detrimental for feeding larvae (Lortzing et al., 2019), and Tetranychus egg extract induces defense mechanisms that reduce female fertility (Ojeda-Martinez et al., 2021).

The modular analysis allowed us to identify the key gene drivers of the processes altered by the oviposition. The detection was based on several statistics that analyzed gene behavior related to the stress and within the network. The regulators were mostly transcription factors, chaperones, and genes involved in protein degradation. The products of these groups of genes are highly connected components that participate in a diverse array of cellular processes (Hahn et al., 2011; Marshall and Vierstra, 2019). *T. urticae* main drivers were involved in secondary metabolite processes such as *SHM4* (AT4G13930) and *IDH5* (AT5G03290) (Proietti et al., 2013; Yoshida and Hisabori, 2014). Other mechanisms associated with molecule trafficking were represented by genes such as *PTR4* (AT2G02020) and *SAR1* (AT2G33120) (Zhang et al., 2011; Komarova et al., 2012). *P. brassicae* response involved TFs such as *HAF01* (AT1G32750) and *E2F3* (AT2G36010) (Magyar et al., 2012; Fina et al., 2017), and pathogen-associated genes like *RIN4* (AT3G25070) and *MKK3* (AT5G40440) (Afzal et al., 2011; Gao et al., 2021). Some of the central genes were also associated with DNA repair processes such as *RAD23D* (AT5G38470) (Farmer et al., 2010).

## Conclusion

The present reanalysis covers transcriptomic studies related to oviposition and egg extract stress of *A. thaliana*. We identified DEGs that encompassed the previously reported and also new DEGs that were overpassed by individual studies. Presentation of the data on a time-lapse scale and the contrasting of different species results, allowed to have a better perspective of the transcriptomic events. Such perspective allowed also the generalization of pathways involved in the response to oviposition. Information on transcriptional events beyond the usually reported defense mechanisms was possible. Key gene regulators of the response to oviposition were also identified. Therefore, this approach increased the strength and sensitivity of the identification of vital oviposition response genes that may be overlooked by isolated studies.

The comparison of DEGs and BPs from a temporal perspective revealed shared and unique components occurring due to eggs/oviposition. The response was dominated by upregulation and the intensity of the gene expression varied for both species. While *P. brassicae* displayed a rich response that reused functions, *T. urticae* induced less and more diverse time-dependent functions. The response to the latter was quickly regulated at 72 h, whereas regulation to the former occurred at twice that time. Earlier events were similar and involved ROS scavenging and defense responses, but after 24 h, both species displayed functional discrepancies. The last time points were dominated by regulatory functions, which help the plant to return to homeostasis. Altogether, the presented results enrich and clarify the knowledge regarding plant response to eggs/oviposition of herbivores. This information can be utilized for multiple stress response engineering.

## Data Availability Statement

All relevant supporting datasets are included in the article and Supplementary Material. The original studies can be found on the following repositories: National Center for Biotechnology Information (NCBI) BioProject database; *T. urticae* RNA-seq (Ojeda-Martinez et al., 2021) under the accession code GSE168993; *P. bassicae* 72 h Ov RNA-seq (Nallu et al., 2018) under the accession code SRP134094; *P. bassicae* 120 h Ov and Ex RNA-seq (Stahl et al., 2020) under the accession code SRP244078; *P. bassicae* 120 h Ov Microarray (Firtzlaff et al., 2016) under the accession code GSE69623; and *P. bassicae* 144 h Ov Microarray (Lortzing et al., 2019) under the accession code GSE114041. Link to PPI networks and steps followed to assemble them: https://www.cbgp.upm.es/files/Network_Analysis.php.

## Author Contributions

ID and MES conceived the research. DO-M performed most of the experimental research. MES, DO-M, and ID participated in the design, acquisition, analysis, and interpretation of the data, and contributed to the final version of the manuscript. All authors contributed to the article and approved the submitted version.

## Conflict of Interest

The authors declare that the research was conducted in the absence of any commercial or financial relationships that could be construed as a potential conflict of interest.

## Publisher’s Note

All claims expressed in this article are solely those of the authors and do not necessarily represent those of their affiliated organizations, or those of the publisher, the editors and the reviewers. Any product that may be evaluated in this article, or claim that may be made by its manufacturer, is not guaranteed or endorsed by the publisher.

## Footnotes

1
http://metascape.org

## Supplementary Material

Supplementary File 1 Supplementary tables.

Supplementary File 2 PRISMA flow diagram.

Supplementary File 3 Unique genes by RNA-seq study.
