# Supplementary material for: Transcriptomic Landscape of Herbivore Oviposition in Arabidopsis: A Systematic Review
Source: Front Plant Sci. 2022 Jan 21;12:772492. doi: 10.3389/fpls.2021.772492 (PMC8815302; doi:10.3389/fpls.2021.772492)
Supplement: Supplementary File 2 — PRISMA flow diagram. [file Table_2.DOCX]

**Identification of studies via other methods**

**Identification of studies via databases and registers**

Records identified from:

GoogleScholar (n = 3150)

Author-specific search (n = 15)

Records explored from:

Databases (n = 500)

**Identification**

Records screened

(n = 500)

Records excluded

(n = 494)

Reports sought for retrieval

(n = 9)

Reports sought for retrieval

(n = 6)

Reports not retrieved

(n = 0)

**Screening**

Reports assessed for eligibility

(n = 9)

Reports excluded:

Reason 1 (n = 5, Non-*A. thaliana* experiments)

Reason 2 (n = 1, Raw data not available)

Reports assessed for eligibility

(n = 6)

Reports excluded:

Reason 1 (n = 1, Controls partially stressed)

Reason 3 (n = 4, Non-*A. thaliana* experiments)

Studies included in review

(n = 5)

Reports of included studies

(n = 5)

*The *T. urticae* data was not published at the time of the analysis

**Included**

*From:*  Page MJ, McKenzie JE, Bossuyt PM, Boutron I, Hoffmann TC, Mulrow CD, et al. The PRISMA 2020 statement: an updated guideline for reporting systematic reviews. BMJ 2021;372:n71. doi: 10.1136/bmj.n71. For more information, visit: <http://www.prisma-statement.org/>
