# Supplementary material for: Transcriptomic Landscape of Herbivore Oviposition in Arabidopsis: A Systematic Review
Source: Front Plant Sci. 2022 Jan 21;12:772492. doi: 10.3389/fpls.2021.772492 (PMC8815302; doi:10.3389/fpls.2021.772492)
Supplement: Supplementary File 4 — Supplementary figures. [file Presentation_1.PPTX]

## Slide 1
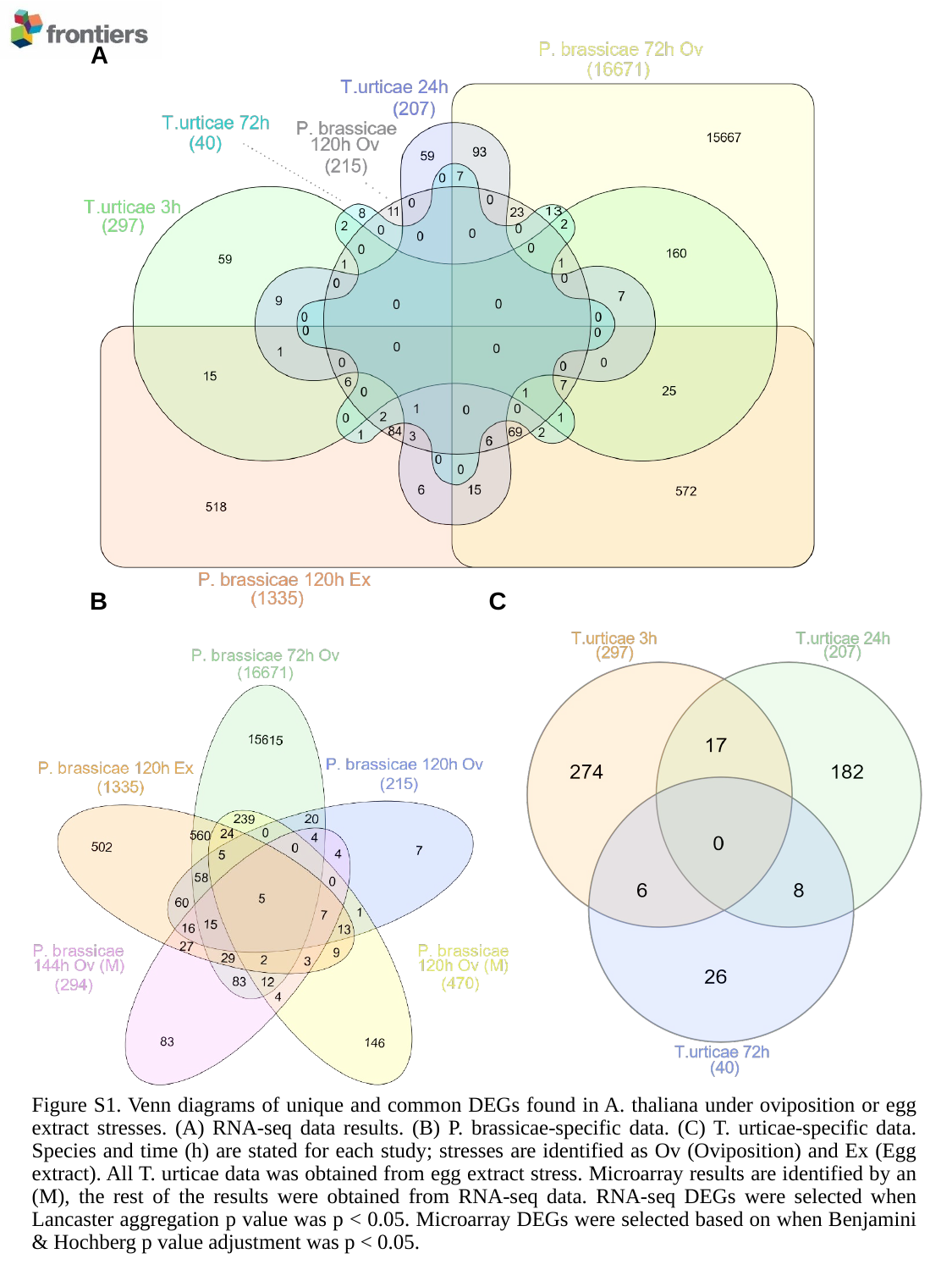

A
B
C
Figure S1. Venn diagrams of unique and common DEGs found in A. thaliana under oviposition or egg extract stresses. (A) RNA-seq data results. (B) P. brassicae-specific data. (C) T. urticae-specific data. Species and time (h) are stated for each study; stresses are identified as Ov (Oviposition) and Ex (Egg extract). All T. urticae data was obtained from egg extract stress. Microarray results are identified by an (M), the rest of the results were obtained from RNA-seq data. RNA-seq DEGs were selected when Lancaster aggregation p value was p < 0.05. Microarray DEGs were selected based on when Benjamini & Hochberg p value adjustment was p < 0.05.

## Slide 2
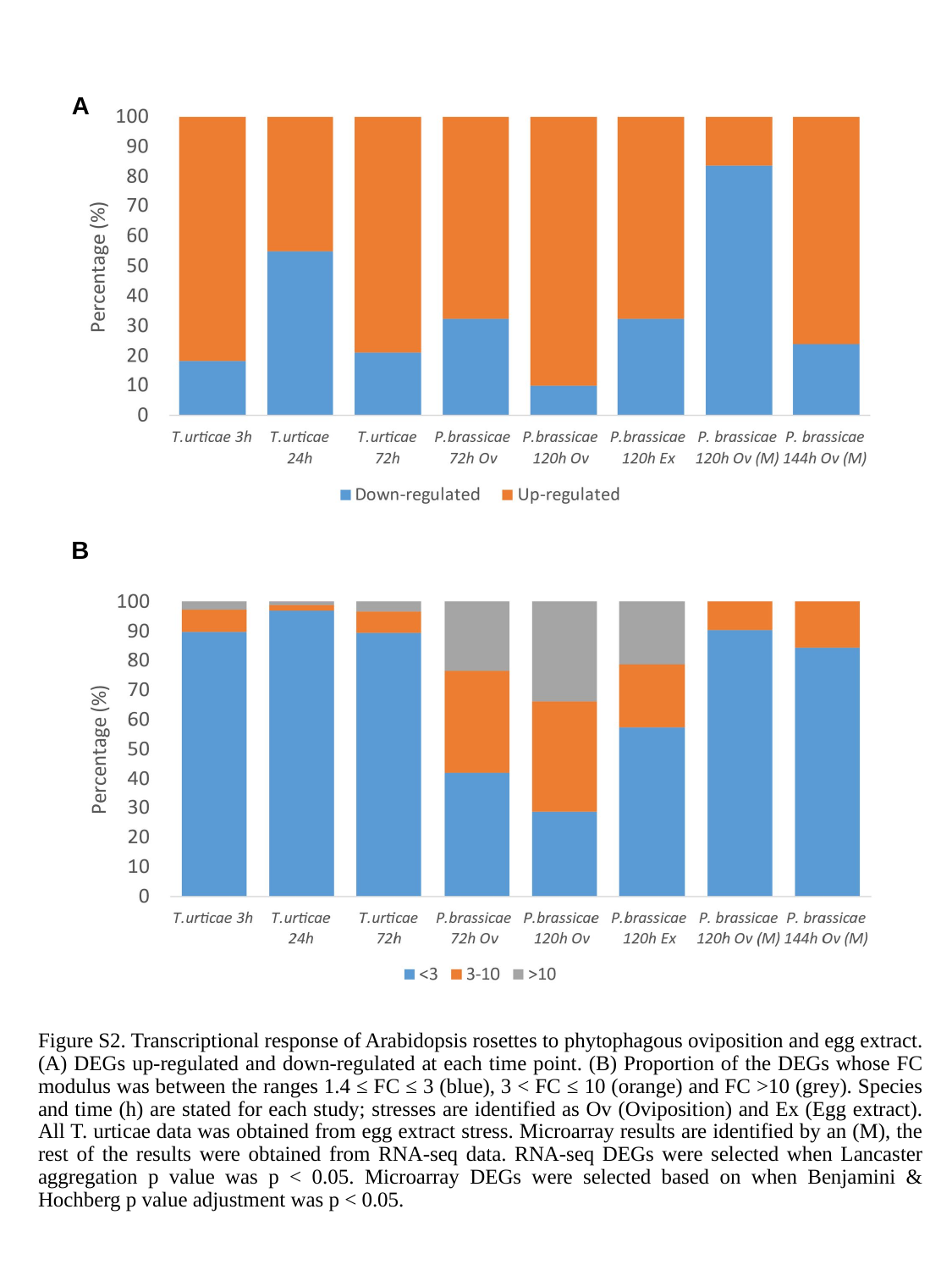

A
B
Figure S2. Transcriptional response of Arabidopsis rosettes to phytophagous oviposition and egg extract. (A) DEGs up-regulated and down-regulated at each time point. (B) Proportion of the DEGs whose FC modulus was between the ranges 1.4 ≤ FC ≤ 3 (blue), 3 < FC ≤ 10 (orange) and FC >10 (grey). Species and time (h) are stated for each study; stresses are identified as Ov (Oviposition) and Ex (Egg extract). All T. urticae data was obtained from egg extract stress. Microarray results are identified by an (M), the rest of the results were obtained from RNA-seq data. RNA-seq DEGs were selected when Lancaster aggregation p value was p < 0.05. Microarray DEGs were selected based on when Benjamini & Hochberg p value adjustment was p < 0.05.

## Slide 3
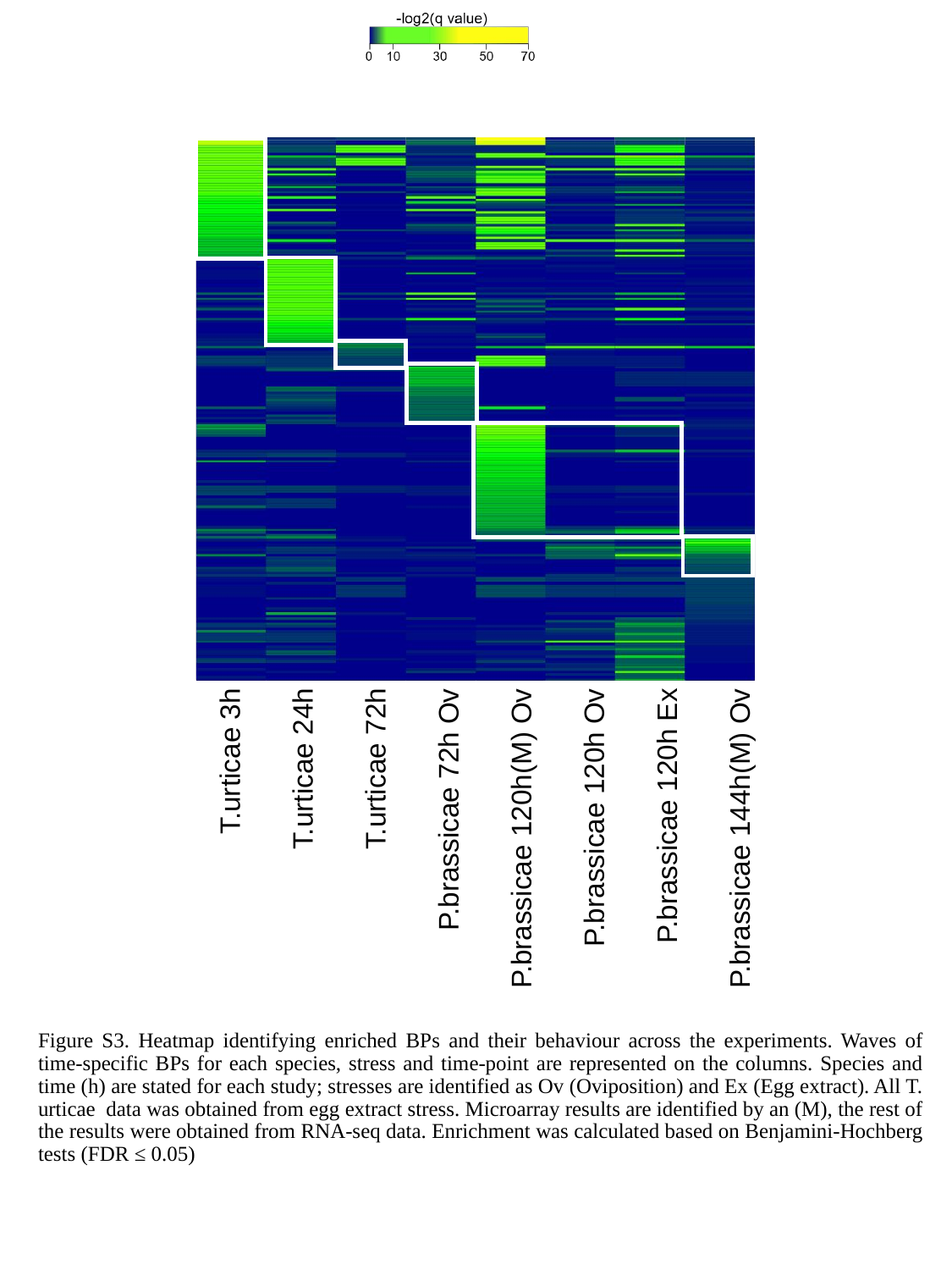

T.urticae 3h
T.urticae 24h
T.urticae 72h
P.brassicae 72h Ov
P.brassicae 120h(M) Ov
P.brassicae 120h Ov
P.brassicae 120h Ex
P.brassicae 144h(M) Ov
Figure S3. Heatmap identifying enriched BPs and their behaviour across the experiments. Waves of time-specific BPs for each species, stress and time-point are represented on the columns. Species and time (h) are stated for each study; stresses are identified as Ov (Oviposition) and Ex (Egg extract). All T. urticae data was obtained from egg extract stress. Microarray results are identified by an (M), the rest of the results were obtained from RNA-seq data. Enrichment was calculated based on Benjamini-Hochberg tests (FDR ≤ 0.05)

## Slide 4
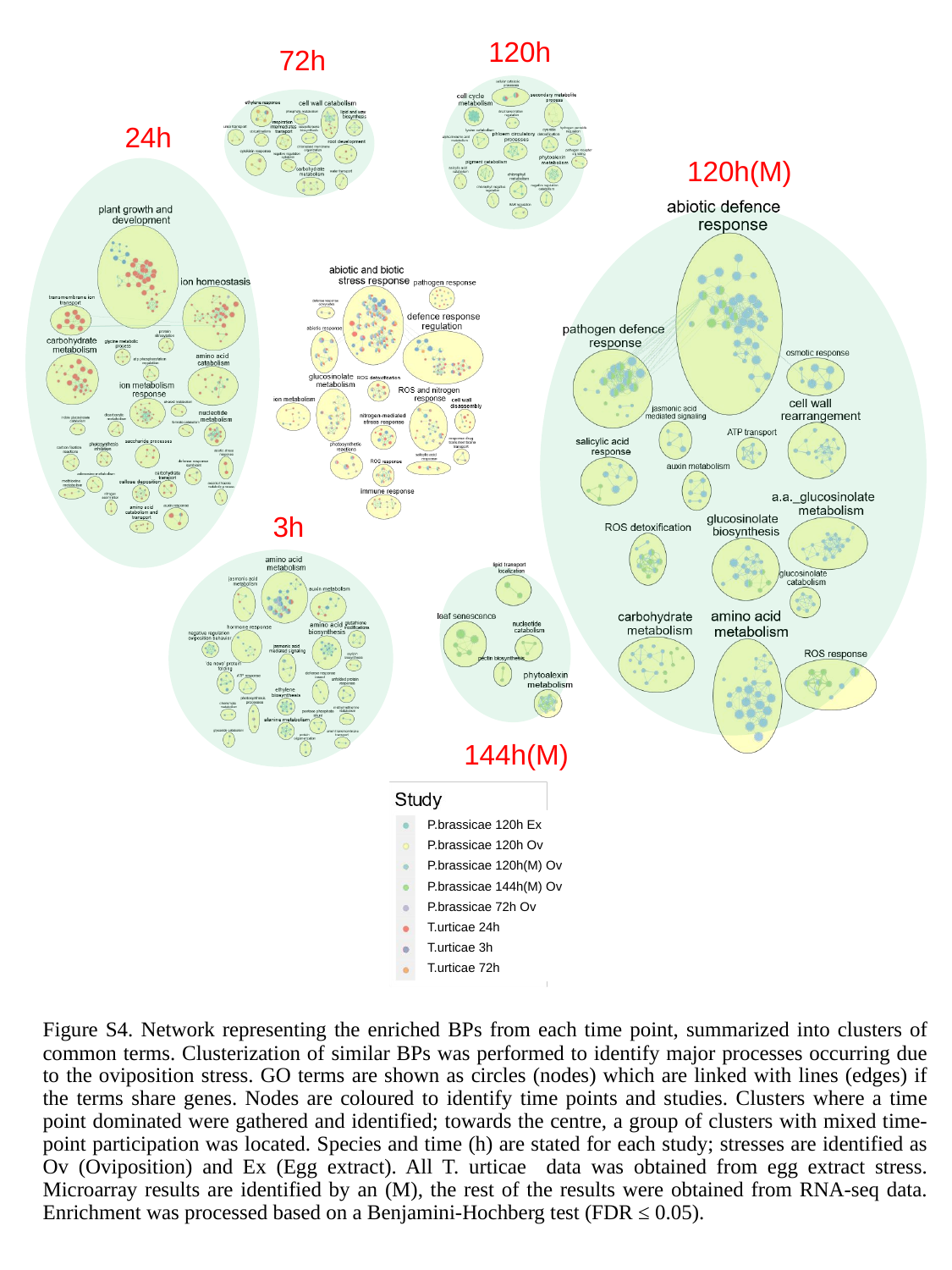

120h
72h
24h
120h(M)
3h
144h(M)
P.brassicae 120h Ex
P.brassicae 120h Ov
P.brassicae 120h(M) Ov
P.brassicae 144h(M) Ov
P.brassicae 72h Ov
T.urticae 24h
T.urticae 3h
T.urticae 72h
Figure S4. Network representing the enriched BPs from each time point, summarized into clusters of common terms. Clusterization of similar BPs was performed to identify major processes occurring due to the oviposition stress. GO terms are shown as circles (nodes) which are linked with lines (edges) if the terms share genes. Nodes are coloured to identify time points and studies. Clusters where a time point dominated were gathered and identified; towards the centre, a group of clusters with mixed time-point participation was located. Species and time (h) are stated for each study; stresses are identified as Ov (Oviposition) and Ex (Egg extract). All T. urticae data was obtained from egg extract stress. Microarray results are identified by an (M), the rest of the results were obtained from RNA-seq data. Enrichment was processed based on a Benjamini-Hochberg test (FDR ≤ 0.05).

## Slide 5
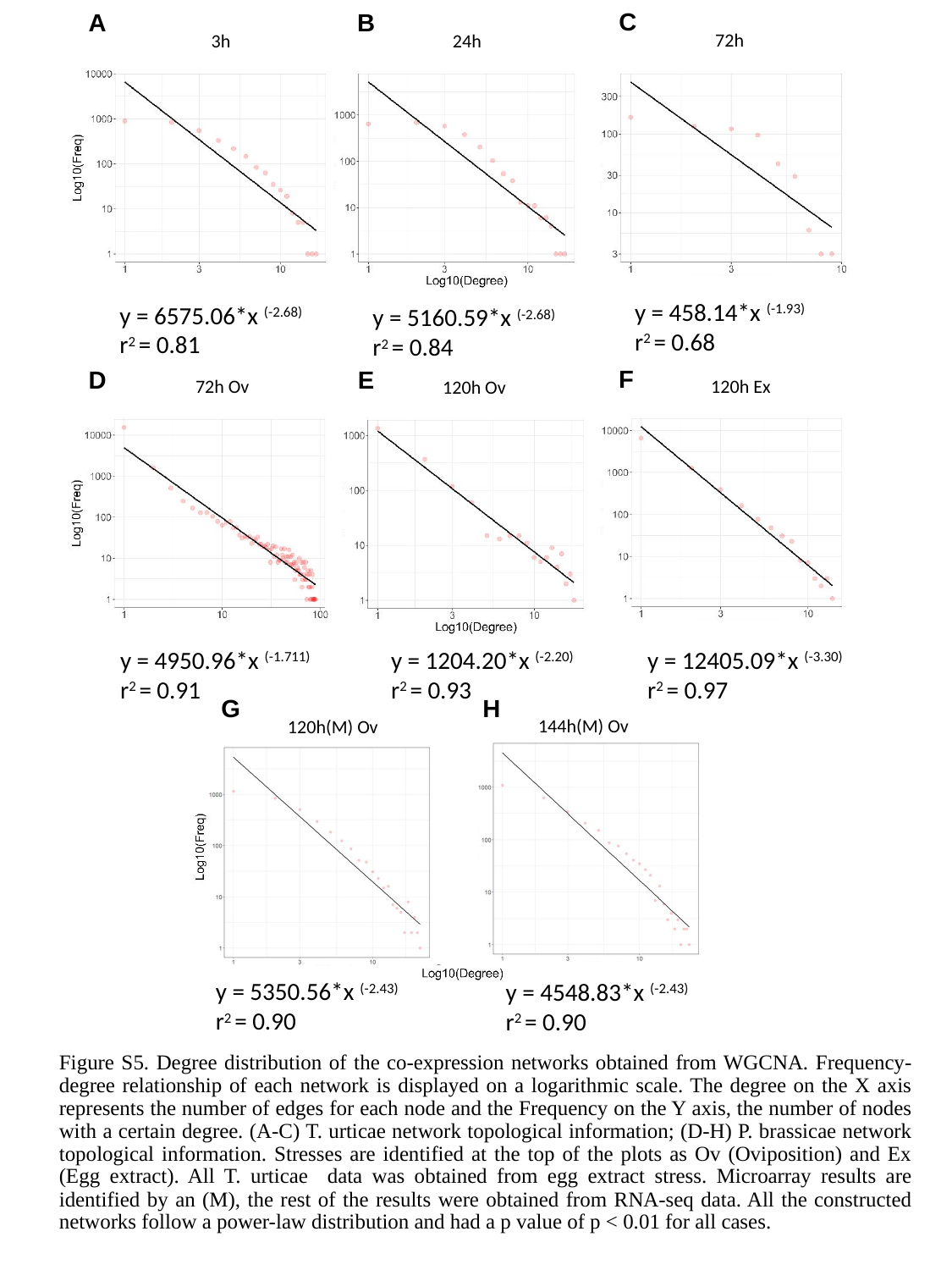

C
A
B
72h
3h
24h
y = 458.14*x (-1.93)
r2 = 0.68
y = 6575.06*x (-2.68)
r2 = 0.81
y = 5160.59*x (-2.68)
r2 = 0.84
F
D
E
72h Ov
120h Ex
120h Ov
y = 4950.96*x (-1.711)
r2 = 0.91
y = 1204.20*x (-2.20)
r2 = 0.93
y = 12405.09*x (-3.30)
r2 = 0.97
H
G
144h(M) Ov
120h(M) Ov
y = 5350.56*x (-2.43)
r2 = 0.90
y = 4548.83*x (-2.43)
r2 = 0.90
Figure S5. Degree distribution of the co-expression networks obtained from WGCNA. Frequency-degree relationship of each network is displayed on a logarithmic scale. The degree on the X axis represents the number of edges for each node and the Frequency on the Y axis, the number of nodes with a certain degree. (A-C) T. urticae network topological information; (D-H) P. brassicae network topological information. Stresses are identified at the top of the plots as Ov (Oviposition) and Ex (Egg extract). All T. urticae data was obtained from egg extract stress. Microarray results are identified by an (M), the rest of the results were obtained from RNA-seq data. All the constructed networks follow a power-law distribution and had a p value of p < 0.01 for all cases.

## Slide 6
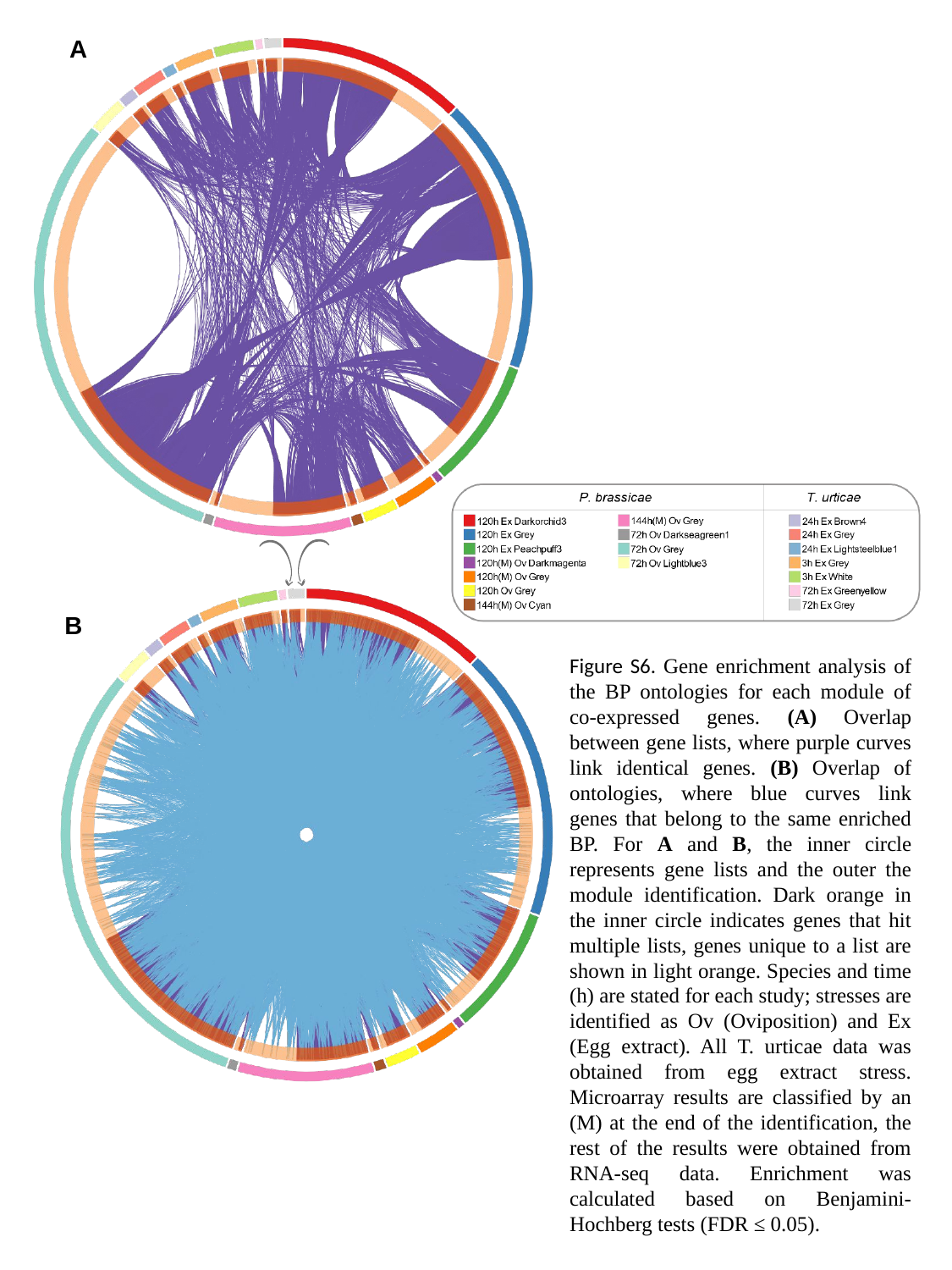

A
B
Figure S6. Gene enrichment analysis of the BP ontologies for each module of co-expressed genes. (A) Overlap between gene lists, where purple curves link identical genes. (B) Overlap of ontologies, where blue curves link genes that belong to the same enriched BP. For A and B, the inner circle represents gene lists and the outer the module identification. Dark orange in the inner circle indicates genes that hit multiple lists, genes unique to a list are shown in light orange. Species and time (h) are stated for each study; stresses are identified as Ov (Oviposition) and Ex (Egg extract). All T. urticae data was obtained from egg extract stress. Microarray results are classified by an (M) at the end of the identification, the rest of the results were obtained from RNA-seq data. Enrichment was calculated based on Benjamini-Hochberg tests (FDR ≤ 0.05).
